# Vapor-based polymers: from films to nanostructures

**DOI:** 10.3762/bjnano.8.221

**Published:** 2017-10-24

**Authors:** Meike Koenig, Joerg Lahann

**Affiliations:** 1 Karlsruhe Institute of Technology (KIT), Institute of Functional Interfaces (IFG), Hermann-von-Helmholtz-Platz 1, 76344 Eggenstein-Leopoldshafen, Germany,; 2 Biointerfaces Institute, University of Michigan (UM), 2800 Plymouth Rd., Ann Arbor, MI 48109, USA

**Keywords:** nanostructures, vapor-based polymers

While the traditional and popular realm of polymer synthesis is the liquid phase, the use of vapor-based techniques to deposit polymers has been met with increasing interest over the past decades. The perhaps most relevant example, the deposition of poly(*p*-xylylenes) via the Gorham process, has been of industrial use in the fabrication of isolating or protective coatings in electronics and biomaterials for many years [1–2]. More recently, vapor deposition polymerization has been extended to a broad variety of reactive polymers [3], additionally using techniques such as plasma-, initiated-, or oxidative chemical vapor deposition polymerization [4–5]. The reason for the ongoing interest in this research field is that, analogue to the deposition of inorganic coatings by chemical vapor deposition, the deposition of polymer coatings from the vapor phase has many advantages over traditional, wet chemistry methods. These advantages result in a variety of benefits for vapor-based polymer coatings and their broad utility.

Due to the absence of solvents, dewetting effects are less pronounced, which together with the fact that small monomers instead of larger polymers are used, enable the highly conformal character of vapor-based coatings. The review article by Moni et al., within this Thematic Series, highlights this feature and discusses how to assess it, as well as its applications [6]. In their research article, Cheng and Gupta present another exemplary application of vapor-borne coatings. Here, a 3D-printed device can be equipped with the desired functionality on its surface, while the bulk material can be chosen independently, according to the requirements of the printing process and the desired mechanical properties [7]. The conformal character of vapor-based coatings can furthermore be used to create nanostructures. Balkan et al. report the formation of coaxial nanotubes by the consecutive deposition of polyaniline and poly(hydroxyethyl methacrylate) on a template with mesoscopic pores [8]. The dissolution of the template results in the desired nanostructures. This combination of a conductive polymer with a hydrogel in a single nanostructure shows potential for the use in humidity sensors.

Without the need for additives or solvents, a high purity of the resulting film is ensured, which is of paramount importance in the area of electronic applications. In this context, the versatility of poly(chloro-*p*-xylylene) in the application of flexible organic electronics is presented in a review article by Marszalek et al. [9]. The absence of small molecule compounds or solvents in CVD films mitigates the risk of potential leakage of hazardous residues from the coating material, which, in turn, often results in superior biocompatibility [10]. This, together with the conformal character of the coating, is of importance in a novel antibacterial catheter introduced by Franz et al. [11] Here, poly(*p*-xylylene), which is deposited via chemical vapor deposition, is used as a top layer above an electro-deposited silver coating, ensuring the prolonged release of antibacterial silver ions.

Another advantage of vapor deposition techniques is the potential of synthesizing copolymers of chemically or functionally distinct monomers [12]. Alternatively, vapor deposition enables coatings of polymers that have only low levels of solubility in solvents. This highlights the importance of vapor deposition techniques in the field of conductive polymers, which are often insoluble and hard to process. Smolin et al. report the deposition of polyaniline using oxidative chemical vapor deposition [13]. A variation of the process parameters influences the quality of the deposited film as the oligomer content or the oxidation state. Another example of polymers with low solubility are fluoropolymers. Christian and Coclite investigated the deposition of fluoroacrylate polymer thin films via initiated chemical vapor deposition and the impact of crosslinking on the mechanical and chemical stability [14].

Vapor-based techniques can also be used to create chemically or topographically structured coatings on various substrates, which is of interest for example in the development of sensors or biomaterials. A general overview of the different techniques used to create structures can be found in the review by H.-Y. Chen [15]. In our own review, on the other hand, we focus on techniques to directly create structures in situ during the vapor deposition [16].

In summary, this Thematic Series highlights the broad utility of polymers deposited from the vapor phase used in the development of novel coating materials for a manifold of applications. We strongly hope that this Thematic Series stimulates further research into new applications of vapor-deposited polymers.

Meike Koenig and Joerg Lahann

Karlsruhe, September 2017
